# Clinical Outcomes of Tube Feeding vs. Hand Feeding in Advanced Dementia

**DOI:** 10.3390/jcm13216535

**Published:** 2024-10-30

**Authors:** Wei Yu Chua, Eng-King Tan

**Affiliations:** 1 Yong Loo Lin School of Medicine, National University of Singapore, Singapore 117597, Singapore; chuaweiyu@u.nus.edu; 2 Department of Neurology, Singapore General Hospital Campus, National Neuroscience Institute, Singapore 308433, Singapore; 3 Neuroscience and Behavioral Disorders, Duke-NUS Medical School, Singapore 169857, Singapore

**Keywords:** dementia, aging, feeding, outcomes, public health

## Abstract

Dementia is a growing public health issue, with the number of cases projected to triple by 2050 as society ages. Although the American Geriatrics Society recommends careful hand feeding over tube feeding for patients with advanced dementia, an increasing proportion of patients are receiving tube feeding. Although decisions regarding tube feeding are often based on the physician, recent studies have shown that tube feeding has significant implications for clinical outcomes and quality of life. Tube feeding is associated with an increased risk of mortality, pneumonia and the use of restraints. Although tube feeding may reduce caregiver burden, it does not improve survival or nutritional status and incurs significant financial costs. Caregivers that hand feed patients often experience stress, particularly in regions where support services are limited. However, there are various strategies available to promote hand feeding which include environmental interventions, mealtime assistance and caregiver training. Although hand feeding is the most comfortable option for patients, the frequency of mealtimes and financial and mental health impact on caregivers requires the physician to conduct a holistic assessment of the patient when deciding on the mode of feeding for patients with advanced dementia.

## 1. Introduction

Dementia is a public health priority and a growing concern for societies worldwide. The number of people suffering from dementia is estimated to triple by 2050 to more than 150 million people worldwide, contributed to by population growth and aging [1]. Alzheimer’s disease, which is the commonest form of dementia, became the seventh leading cause of death in the United States in 2022 [2]. In patients with advanced dementia, the decline in cognitive ability interferes with basic activities of daily living, such as eating [3]. A study of 186,835 nursing home residents in America found that more than one-third of severely cognitively impaired residents have feeding tubes [3]. In patients with advanced dementia, the options available for feeding include careful hand feeding and tube feeding (nasogastric tube (NGT) or percutaneous endoscopic gastrostomy (PEG)) [4]. The American Geriatrics Society consensus statement does not recommended tube feeding for older adults with advanced dementia and recommends careful handfeeding [4]. However, across the developed world, the caregiver support ratio (CSR) is projected to decrease significantly. In Europe, the CSR is projected to fall from 4.68 in 2020 to 2.09 in 2050 [5], with countries in Asia reporting similar figures [6]. Therefore, we examined the clinical outcomes of tube feeding and careful hand feeding to provide recommendations for physicians of patients with advanced dementia.

## 2. Discussion

In early stages of advanced dementia, dysphagia undergoes a prolonged oral phase characterized by reduced lingual movement and a delayed swallowing reflex [7]. As dementia worsens, there is increased difficulty in bolus preparation, airway clearance, upper esophageal sphincter opening, and visible aspiration occurs [8]. In severe stages, swallowing difficulties worsen, and patients may experience swallowing apraxia [9]. In patients with severe dysphagia, tube feeding is a pivotal form of artificial nutrition. However, the use of tube feeding is associated with agitation, the use of physical and chemical restraints, hospitalization from tube-related complications and pressure ulcers [4]. Although it is uncomfortable for the patient, the decision to tube feed is often due to concerns about dysphagia and poor nutrition [10]. However, a recent meta-analysis by Lee et al. found that PEG feeding is associated with increased mortality rate (OR 1.79, 95% CI: 1.04–3.07; *p* = 0.03) and pneumonia (OR 3.56, 95% CI: 2.32–5.44, *p* < 0.001) complications, and it does not prolong survival days or improve nutritional status in patients with advanced dementia [11]. Furthermore, financial considerations may influence the decision to tube feed a patient with dementia who can no longer eat independently [12]. A 6-month cost comparison study by Mitchell et al. found that although patients on PEG feeding require less expensive nursing home care (USD 2379 vs. USD 4219), greater expenses are incurred due to feeding tube placement and the acute management of complications (USD 6994 vs. USD 959) [12].

Compared to the West, many Asian countries including China [13], Taiwan [14], and Singapore [15], favor the use of NGT as the main mode of tube feeding. The decision to insert either a PEG or an NGT depends on the perspective of the caregiver and medical staff. In Asian society, patients requiring artificial nutrition are less willing to undergo additional operation and prefer to maintain their body’s integrity [15]. However, these beliefs are less prevalent in the West, where PEG is the preferred option for tube feeding in patients with advanced dementia. This may be attributed to the longer duration of use and better patient comfort with PEG compared to NGT, where more frequent replacements are needed [16]. Although studies on PEG are largely confined to the West, there are only a few studies investigating the outcomes of NGT compared to careful hand feeding in patients with advanced dementia. Chou et al. analyzed 39 and 130 advanced dementia patients receiving careful hand feeding and NGT feeding, respectively [17]. The study in Taiwan found no significant differences in 1-year mortality or hospitalization and a nonsignificant trend of increased risk of pneumonia was observed in the nasogastric tube group [17]. A larger study in Hong Kong by Yuen et al. comprising 300 and 464 advanced dementia patients receiving careful hand feeding and nasogastric tube feeding, respectively, found that nasogastric tube feeding is associated with a higher pneumonia risk, particularly in patients with both dysphagia and behavioral feeding problems. Compared to Chou et al., Yuen et al. had a much larger sample size and included the severity of dysphagia as a possible confounder in the analysis. In the study by Yuen et al., the decision to opt for nasogastric feeding or careful hand feeding was based on a consensus made between the physician and the family [18]. In the cohort of patients receiving nasogastric tube feeding, a significantly higher proportion had experienced a previous aspiration pneumonia episode (64.6% vs. 35.4%, *p* = 0.02) than patients on careful hand feeding, although the Charlson Comorbidity Index (5.9 vs. 5.9, *p* = 0.97) was similar in both groups [18]. As Yuen et al. excluded patients who had received tube feeding prior, this highlights that a history of aspiration pneumonia could be the impetus for caregivers to seek the insertion of a nasogastric tube [18]. The decision to initiate nasogastric tube feeding must be made following a holistic assessment of the patient, and physicians must explore the reasons behind the decision to start using a nasogastric tube.

A study by Sharma et al. in Malaysia on caregiver experiences with hand feeding found that many caregivers reported high distress, which was positively correlated with dementia severity [19]. In addition to frequent mealtimes, caregivers often have numerous responsibilities, including personal care, managing medications, and dealing with challenging behaviors. Furthermore, 70% of caregivers reported that they did not have easy access to support and do not know where to seek assistance from [19]. A study by Kitamura et al. in Japan emphasized the importance of support groups, respite care, and counselling in reducing caregiver stress [20]. In addition, the introduction of home-visit nurses through the Japanese Comprehensive Strategy to Accelerate Dementia Measures, has helped to provide caregivers with a deeper understanding of dementia and appropriate care for dementia patients, reducing caregiver anxiety and embarrassment [20]. For families with suitable resources, foreign domestic workers can be engaged as caregivers [21]. In a study conducted in Singapore by Yuan et al., having a foreign domestic worker helped reduce depressive symptoms of the main caregiver through physical and psychological support in the daily care of the patient with dementia [21].

Each modality of feeding has its own set of risks and benefits. PEG requires less feeding time but is associated with increased mortality rate, pneumonia and costs incurred from feeding tube placement and the management of complications [11]. The use of NGT feeding does not impact mortality rate and may slightly increase the risk of pneumonia (13). However, it can be performed in an outpatient setting without a significant financial burden on caregivers and is less invasive compared to PEG. Although careful hand feeding is the recommended mode of feeding, frequent mealtimes coupled with other caregiving responsibilities has a significant impact on the mental health of caregivers [19]. Therefore, in patients with dementia, careful hand feeding should be the standard of care. However, depending on individual circumstances, family support and patient factors, tube feeding can be considered (Table 1).

There are various strategies available to promote hand feeding in patients with advanced dementia [22]. The strategies include environmental interventions, mealtime assistance and caregiver training. The use of piano and nature music was found to significantly reduce mealtime agitation [23] and tableware contrast manipulation was found to improve food intake by 25% [24]. Mealtime assistance interventions consisting of starting a conversation about the food and the use of structured verbal cues were found to encourage patients to eat for a longer duration [25,26]. Lastly, training caregivers in hand feeding techniques and feeding behaviors among patients with dementia was also found to improve the wellbeing of caregivers and the attention span of patients during meal times [27] (Table 2).

## 3. Limitations and Further Directions

Due to the limited number of studies examining each mode of feeding, we were unable to conduct a subgroup analysis between different types of tube feeding and careful hand feeding. There is a distinct geographical variation in the modality of tube feeding. All the studies on PEG included in the meta-analysis by Lee et al. were conducted in the West [11], whereas all the studies on NGT were conducted in Asian countries. Furthermore, studies did not define the nature of careful hand feeding or the feeds provided for tube feeding. The caregiver support ratio is only applicable to free living individuals and differs from patients living in nursing homes. A study by Harrington et al. found that the patients per nurse ratio varies from nine to twenty patients, and this could potentially affect the choice of nutrition [28]. Differences in practices in different healthcare systems (public vs. private) may potentially affect the choice of nutrition as well.

Future studies can consider conducting randomized control trials to examine the impact of various feeding types on patients as well as caregivers. Long-term health economic studies are required to evaluate the cost effectiveness of each modality of feeding. There is a need to raise the vigilance of aspiration pneumonia and other complications for feeding in chronic neurological disorders like dementia.

## 4. Conclusions

With the expected increase in patients with advanced dementia and decreasing number of caregivers, tube feeding is expected to become increasingly common. Although careful hand feeding remains the ideal mode of feeding, the declining CSR ratio will present physicians and family members with a dilemma regarding the mode of feeding. To address this key issue, more randomized control trials on clinical outcomes, cost effectiveness and quality of life in patients and caregivers for each approach is required. Further studies to develop new care strategies that can promote manual feeding in patients with dementia are required as well. There is a need to raise the vigilance of aspiration pneumonia and other complications for feeding in chronic neurological disorders like dementia.

## Figures and Tables

**Table 1:** Pros and cons of different feeding types in patients with dementia.

	Pros	Cons
Percutaneous Endoscopic Gastrostomy (PEG)	Requires less feeding time Lower cost of daily care	Increased risk of mortality and pneumonia Higher costs incurred from feeding tube placement and management of complications Does not prolong survival days and nutritional status in patients with advanced dementia
Nasogastric Tube (NGT)	Insertion of NGT can be performed in outpatient clinic or in the community Less financial burden compared to PEG Alternative source of feeding in patients with severe dysphagia	No mortality benefit Slight increased risk of pneumonia
Hand feeding	Most comfortable for patient Reduced agitation, use of physical and chemical restraints Reduction in hospitalization from tube-related complications and pressure ulcers	Frequent mealtimes Negative impact on mental health of caregivers Higher costs of daily care

**Table 2:** Strategies available to promote hand feeding in patients with dementia.

	Strategy	Benefits
Environmental Interventions	Music (piano and/or nature sounds) Tableware contrast manipulation	Reduces mealtime agitation Improves food and fluid intake
Mealtime Assistance Interventions	Conversation with patient regarding food Structured verbal cues with positive reinforcement	Encourages patient to eat for longer duration
Caregiver Training	Courses on hand feeding techniques Courses on etiology, behavior and management of dementia	Increases total eating time of patients Improved knowledge, behavior and positive attitude towards patient

## Data Availability

Not applicable.
